# Is the Deliberate Practice View Defensible? A Review of Evidence and Discussion of Issues

**DOI:** 10.3389/fpsyg.2020.01134

**Published:** 2020-08-18

**Authors:** David Z. Hambrick, Brooke N. Macnamara, Frederick L. Oswald

**Affiliations:** ^1^Department of Psychology, Michigan State University, East Lansing, MI, United States; ^2^Department of Psychological Sciences, Case Western Reserve University, Cleveland, OH, United States; ^3^Department of Psychological Sciences, Rice University, Houston, TX, United States

**Keywords:** deliberate practice, expertise, talent, skill, individual differences

## Abstract

The question of what explains individual differences in expertise within complex domains such as music, games, sports, science, and medicine is currently a major topic of interest in a diverse range of fields, including psychology, education, and sports science, to name just a few. Ericsson and colleagues’ *deliberate practice view* is a highly influential perspective in the literature on expertise and expert performance—but is it viable as a testable scientific theory? Here, reviewing more than 25 years of Ericsson and colleagues’ writings, we document critical inconsistencies in the definition of deliberate practice, along with apparent shifts in the standard for evidence concerning deliberate practice. We also consider the impact of these issues on progress in the field of expertise, focusing on the empirical testability and falsifiability of the deliberate practice view. We then discuss a multifactorial perspective on expertise, and how open science practices can accelerate progress in research guided by this perspective.

## Is the Deliberate Practice View Defensible? A Review of Evidence and Discussion of Issues

Not infrequently, a single theoretical perspective becomes extremely influential in an area of scientific inquiry, shaping the trajectory of research in the field for years or even decades. More than 25 years ago, K. Anders Ericsson and colleagues proposed what has arguably become the most influential theoretical perspective in the scientific literature on expertise and expert performance. In a pivotal *Psychological Review* article, Ericsson et al. (1993) theorized that expert performance reflects a long period of *deliberate practice*, which they stated “includes activities that have been specially designed to improve the current level of performance” (p. 368). In studies of violinists (Study 1) and pianists (Study 2), Ericsson et al. (1993) operationally defined deliberate practice as “practice alone” with the goal of improving performance. The most accomplished musicians reported having accumulated an average of around 10,000 h of practice alone by early adulthood, which was thousands of hours more than the averages for this measure of practice for less accomplished groups.

Applying their framework to several domains of expertise, Ericsson et al. (1993) concluded that “individual differences in ultimate performance can largely be accounted for by differential amounts of past and current levels of practice” (p. 392). They further explained:

[H]igh levels of deliberate practice are necessary to attain expert level performance. Our theoretical framework can also provide a sufficient account of the major facts about the nature and scarcity of exceptional performance. Our account does not depend on scarcity of innate ability (talent) (Ericsson et al., 1993, p. 392).

Reiterating this perspective, Ericsson et al. (2007) stated that “[i]t is possible to account for the development of elite performance among healthy children without recourse to unique talent (genetic endowment)—excepting the innate determinants of body size” (p. 4). And writing in the *Harvard Business Review*, Ericsson et al. (2007) explained, “Our research shows that even the most gifted performers need a minimum of 10 years (or 10,000 h) of intense training before they win international competitions” (p. 119).

Ericsson and colleagues’ perspective, which we refer to as the *deliberate practice view*, has had a monumental impact on expertise research. As of this publication, the Ericsson et al. (1993) article has been cited nearly 11,000 times in a wide range of literatures, and there have been nearly 200 theses and dissertations on deliberate practice in universities around the world. As portrayed in popular press books such as Malcolm Gladwell’s (2008) bestseller *Outliers: The Story of Success* and Daniel Coyle’s (2009) *The Talent Code*, Ericsson and colleagues’ research has also had a profound influence on the public’s thinking about the origins of expertise. Taking his inspiration from Ericsson et al.’s (1993) findings, Gladwell wrote that “10,000 h is the magic number of true expertise” (p. 11). In his own popular press book, *Peak: Secrets from the New Science of Expertise*, Ericsson wrote, “Deliberate practice can open the door to a world of possibilities that you may have been convinced were out of reach. Open that door” (Ericsson and Pool, 2016, p. 179).

We credit and commend Ericsson and colleagues for their highly influential work. However, here we will discuss what we believe are serious concerns with whether the deliberate practice view is viable as a *scientific theory—*that is, whether it is empirically testable and falsifiable. [For a similar type of review, see Gottfredson’s (2003) critique of Sternberg’s practical intelligence theory; Sternberg et al. (1995)]. Before doing so, however, we note two uncontroversial claims about expertise, by which we simply mean a person’s measurable (i.e., quantifiable) level of performance in a domain. First, as Ericsson and colleagues have emphasized (e.g., Ericsson, 2006), *expertise is acquired gradually.* In other words, people are not *literally* born as experts, innately endowed with the type of specialized knowledge that underpins high-level skill in activities like hitting a golf ball, solving math equations, playing an instrument, or choosing a move in a chess game. Domain-specific knowledge and skill can only be acquired gradually over time through some form of training.

The second uncontroversial claim is that training can lead to large, even massive, improvements in people’s level of expertise (i.e., domain-relevant performance). This point was amply illustrated by some of Ericsson and colleagues’ earliest research. For example, in a classic study, Ericsson et al. (1980) showed that after more than 200 h of training, a college student improved his performance in a random digit memorization task from a typical 7 digits to 79 digits (the world record is currently an astounding 547 digits)^1^. In short, notwithstanding the issues raised in this article, Ericsson and colleagues’ deliberate practice view has important value in society, serving as a useful reminder to the layperson that training of some form is necessary to achieve a high level of performance in a domain.

**FIGURE 1:**
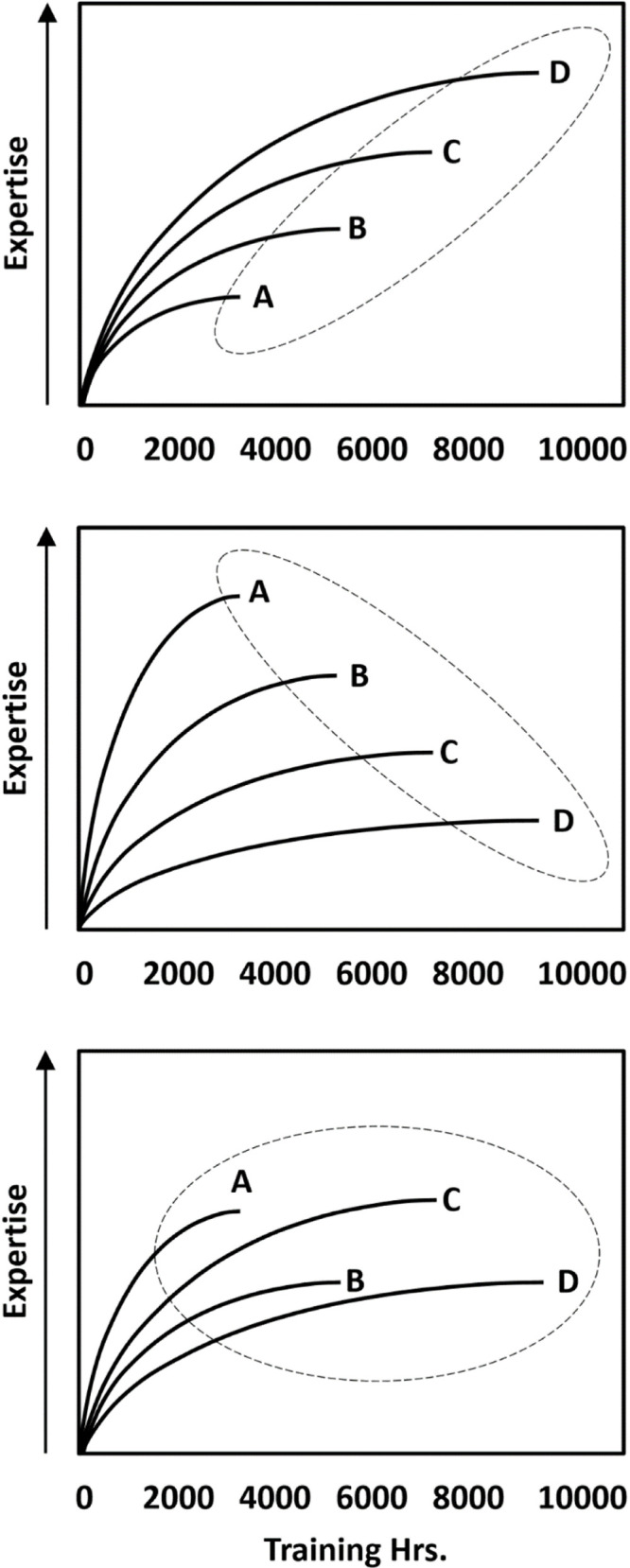
Schematic illustration of a positive (top panel), negative (middle panel), and zero (bottom panel) between-person correlation (as illustrated by the dashed ellipses) between training hours and expertise, assuming a positive relationship between the variables within individuals (as represented by the learning curves for four hypothetical individuals).

The controversial question in research on expertise is not whether some form of training is necessary to explain intraindividual (i.e., within-person) increases in expertise (it must be), or whether these increases can be massive (they can be). Rather, the controversial question is the extent to which interindividual (i.e., between-person) differences in accumulated amount of training explain interindividual differences in expertise (for a discussion of the distinctions between interindividual and intraindividual variability, see Molenaar et al., 2003). In statistical terms, what is the direction (and the magnitude) of the correlation between expertise and accumulated amount of training? Somewhat counterintuitively, as Figure 1 illustrates, the necessity of training to explain intraindividual increases in expertise has no direct implication for the answer to this question. That is, taking as a given that the relationship between training and expertise is positive within individuals, the correlation between training and expertise *between* individuals could be positive (top panel), indicating higher levels of performance for individuals who have engaged in more training; negative (middle panel), indicating lower levels of performance for individuals who have engaged in more training; or zero (bottom panel).

What, then, is the correlation between deliberate practice and expertise *across* individuals? The first step in attempting to answer this question is to operationalize deliberate practice—that is, to develop measures of deliberate practice based on the definition of the construct. Unfortunately, as we document in this article, there remains a great deal of confusion about the definition of deliberate practice, despite more than 25 years of research on the topic. As Ericsson and colleagues themselves recently noted: “It has been common for scientists to be confused about the definition of DP (deliberate practice)” (Dearani et al., 2017, p. 1333). Here, we discuss possible sources of this continued confusion, the confusion around the measurement of deliberate practice that has resulted (e.g., inclusion criteria for meta-analyses), and the impact of this confusion on the science of expertise. We conclude this article with thoughts on how to advance the scientific study of expertise and expert performance. For a companion presentation to this article, visit https://osf.io/buqsk/.

## What Is Deliberate Practice?

It is undoubtedly the case that different types of domain-relevant activities vary in their importance for developing expertise. For example, training under a qualified golf instructor is almost certainly more beneficial for improving golf skill than mindlessly hitting practice balls at a driving range. In their article on deliberate practice, Ericsson et al. (1993) distinguished among three forms of domain-specific experience. They described *work* as engagement in activities for external rewards (e.g., music performances, sports competitions), *play* as participating in activities for pleasure (e.g., playing a sport with friends for recreation), and *deliberate practice* as a “highly structured activity, the explicit goal of which is to improve performance” (Ericsson et al., 1993, p. 368).

**FIGURE 2:**
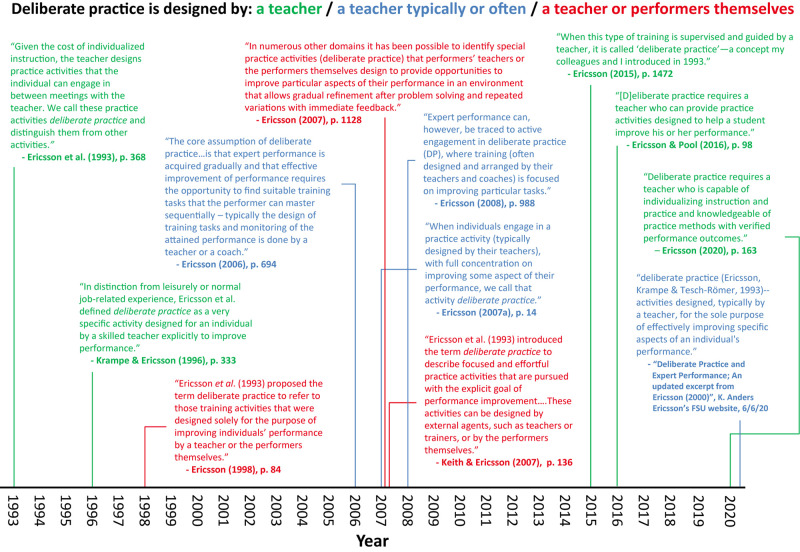
Descriptions of deliberate practice from Ericsson’s writings (1993 – 2020). Adapted from Macnamara and Hambrick (2020) with permission of Springer Nature; this figure can also be found at https://osf.io/buqsk/.

However, Ericsson and colleagues have been inconsistent on critical elements of the definition of deliberate practice, and consequently it has been unclear what activities do and do not qualify as deliberate practice. For example, Ericsson et al. (1993) stated that “the teacher designs practice activities that the individual can engage in between meetings with the teacher” (p. 368). A few years later, however, Ericsson (1998) stated that “Ericsson et al. (1993) proposed the term deliberate practice to refer to those training activities that were designed solely for the purpose of improving individuals’ performance by a teacher *or the performers themselves”* (p. 84, emphasis added). This latter statement indicated that deliberate practice, as Ericsson et al. (1993) originally defined the term, encompasses a broader range of activities than just teacher-designed practice. Yet, as shown in Figure 2, in subsequent articles, Ericsson and colleagues were inconsistent on this critical point, sometimes indicating that deliberate practice must be designed by a teacher (e.g., Ericsson, 2015), but other times stating that it can be designed by teachers or the “performers themselves” (e.g., Keith and Ericsson, 2007). If deliberate practice *must* be designed by a teacher, then presumably it cannot also be designed by performers themselves.

As another example of definitional confusion, it is unclear from Ericsson and colleagues’ writings whether deliberate practice must be a solitary activity, or whether it can also be a group/team activity. Citing research on team sports (Helsen et al., 1998), Ericsson (2006) observed that “the amount of time spent in team-related deliberate practice activities correlates reliably with skill level in team sports” (p. 695). It seems clear from this observation that there can be team deliberate practice. Recently, however, Ericsson and Harwell (2019) indicated that this is *not* the case, commenting that “it is important to point out that organized team training may be quite effective in improving performance, but it does not meet all the criteria for deliberate practice” (p. 6). This is another apparent shift in the definition of and criteria for deliberate practice, creating still more confusion about what the “correct” definition is. In short, it is unclear what activities do and do not qualify as deliberate practice.

This confusion surrounding the definition of deliberate practice is not a minor matter—it directly impacts how deliberate practice is measured in empirical studies and what evidence (i.e., effect sizes) should be included in a meta-analysis. These decisions, in turn, directly impact the evaluation of the deliberate practice view: whether evidence is concluded to support the view or not. Certainly, definitions of theoretical constructs can and do evolve over time as science progresses, but the shifts in the definition of deliberate practice reflected in Figure 2 do not appear to reflect this sort of progression. There are meaningful changes even over short spans of time (e.g., compare Keith and Ericsson’s, 2007, description of deliberate practice with Ericsson’s, 2006). Confusion around the definition of deliberate practice persists in the expertise literature, even as researchers are attempting to investigate the deliberate practice view.

### Challenges to the Deliberate Practice View

Notwithstanding this confusion over the definition of deliberate practice, there have been numerous attempts to test the deliberate practice view. One of the first noteworthy tests came from a study of chess expertise by Gobet and Campitelli (2007), who administered a questionnaire to 90 members of a Buenos Aries chess club to assess lifetime engagement in deliberate practice and tournament chess rating. The self-reported amount of deliberate practice (hours of studying alone plus hours of group practice) correlated positively and moderately with chess rating (*r* = 0.42). This is a sizeable correlation by psychological standards—a “medium” effect size in Cohen’s (1992) widely used classification scheme (i.e., *r* = 0.10, small; *r* = 0.30, medium; *r* = 0.50, large). However, this finding also challenges the deliberate practice view because it means that deliberate practice left a large amount of the total variance in chess ratings *un*explained. To be exact, a correlation of *r* = 0.42 between two variables indicates that one variable explains 18% of the variance in the other variable (i.e., *r* = 0.42 × 100 = 18%). In the present case, it must be assumed that some of this unexplained variance reflects random measurement error, because neither a measure of deliberate practice nor a measure of performance can be assumed to be perfectly reliable (we discuss this issue further below). However, the correlation was not large enough to suggest that, even after taking this psychometric artifact into account, participants at similar levels of chess skill would have reported similar amounts of deliberate practice. Instead, it suggests that the chess players varied substantially in the amount of deliberate practice they required to reach a given level of skill. Indeed, according to the data, they did: As Gobet and Campitelli (2007) described in their article, the self-reported estimate of number of hours of deliberate practice required to reach “master” status in their sample ranged from 3,016 to 23,608 h—a difference of nearly a factor of 8. The implication is that although deliberate practice clearly contributes to individual differences in chess expertise, other factors must contribute as well, as we discuss further in the final section of this article.

In our own first effort to test the deliberate practice view (Hambrick et al., 2014b), we reanalyzed results from expertise studies in the domains of chess and music. Our specific goal was to test Ericsson et al.’s (1993) aforementioned claim that “individual differences in ultimate performance can *largely be accounted for* by differential amounts of past and current levels of practice” (p. 392, emphasis added). We identified six studies of chess and eight studies of music that reported a correlation between a measure of deliberate practice and performance. The average correlation was *r* = 0.49 for chess and *r* = 0.43 for music before applying the standard psychometric correction for measurement error variance (unreliability) of the constituent measures. For deliberate practice, we assumed a reliability coefficient of 0.80 based on information we could find about the reliability of this variable, as well as on Tuffiash et al.’s (2007) statement that “self-report practice estimates repeatedly from experts in sports and music have reported test-retest reliabilities at or above 0.80” (p. 129) and Ericsson’s (2013) statement that “[t]he collected reliability of cumulated life-time practice at different test occasions in large samples has typically been found to range between 0.7 and 0.8” (p. 534). For music performance, we used reliability estimates from the studies, or if not reported, from studies that collected similar performance measures. The average amount of reliable variance in expertise explained by deliberate practice was 34% for chess and 29.9% for music. This is a substantial amount of variance, but it is not enough to support the claim that deliberate practice largely accounts for individual differences in expertise. This claim implies that deliberate practice should at least explain *most* of the variance in expertise, and evidence suggests it does not.

Subsequently, we set out to test the importance of deliberate practice as a predictor of individual differences in expertise, by way of a formal and comprehensive meta-analysis (Macnamara et al., 2014; see also Macnamara et al.’s, 2018, corrigendum for the article), ultimately reviewing over 11,000 articles. Ericsson et al. (1993) explained that deliberate practice “includes activities that have been specially designed to improve the current level of performance” (p. 368). Accordingly, we defined deliberate practice as structured activities designed to improve performance in a domain; and given Ericsson and colleagues’ inconsistency on whether a teacher is required to design deliberate practice, we decided to include both teacher- and performer-designed activities. Identifying 88 studies, we found that deliberate practice explained 14% of the variance in performance overall, and 24% for games, 23% for music, 20% for sports, 5% for education, and 1% for professions. We also determined that deliberate practice left more of the variance in performance unexplained than it explained, across a range of possible values for measurement reliability. We concluded that the “amount of deliberate practice—although unquestionably important as a predictor of individual differences in performance from both a statistical and a practical perspective—is not as important as Ericsson and his colleagues have argued” (Macnamara et al., 2014, p. 1615).

In a later meta-analysis that focused on sports (Macnamara et al., 2016b), we found that the contribution of deliberate practice to sports performance varied by skill level: Among elite athletes (e.g., national-level and above), deliberate practice explained only 1% of the performance variance. Although it must be assumed that range restriction (another psychometric issue) would limit the deliberate practice-performance correlation when considering only elite performers, it is critical to note that it was Ericsson et al. (1993) themselves who stated that deliberate practice is *still* an important predictor of performance differences at the elite level. In their own words, “Individual differences, *even among elite performers*, are closely related to assessed amounts of deliberate practice” (Ericsson et al., 1993, p. 363, emphasis added). This finding from the Macnamara et al. (2016b) meta-analysis on sports is inconsistent with this claim. Furthermore, we found the relationship between deliberate practice and performance to be very similar whether the practice activities were solitary or in a group (see Macnamara et al., 2016b).

In a commentary on our meta-analysis (Macnamara et al., 2014), Ericsson (2014a) rejected 87 of the 88 studies that we included in our meta-analysis, claiming that we included studies that “violated [their] criteria for deliberate practice” (p. 2). However, in doing so, Ericsson (2014a) rejected numerous studies that he himself had previously used to explicitly argue for the importance of deliberate practice (see quotations from Ericsson’s writings in Table 1). Thus, by any reasonable account, the standard for evidence concerning deliberate practice had shifted dramatically. Most perplexingly, in applying this new standard for evidence, Ericsson rejected several of his own studies of deliberate practice (e.g., Duffy et al., 2004; Tuffiash et al., 2007; Duckworth et al., 2011), seeming to undermine the case he had for decades been attempting to make for the importance of deliberate practice. Ericsson did not acknowledge that he had once used these studies he was now rejecting to argue for the importance of deliberate practice. This evaluation of evidence challenging the deliberate practice view seems indefensible.

**TABLE 1:** Examples of studies that Ericsson rejected for violating his criteria for deliberate practice but previously used to argue for the importance of deliberate practice.

Study rejected by Ericsson (2014a) for violating his criteria for deliberate practice	Previous use of the same study by Ericsson and colleagues to argue for the importance of deliberate practice
Hodges and Starkes (1996)^1^	“Several studies and reviews have since found a consistent relation between performance and amount and quality of **deliberate practice**…in sports (…Hodges and Starkes, 1996…).” - Ericsson (1998, p. 87)
Helsen et al. (1998)^1^	“Research conducted in several domains such as…sports (Helsen, Starkes, and Hodges, 1998…) suggests that the amount of accumulated **deliberate practice** is closely related to an individual’s attained level of performance.” - Keith and Ericsson’s (2007, p. 136)
Duffy et al. (2004)^2^	“The engagement of the dart-related activities differed between groups for three types, namely playing in league darts, solitary practice and total **deliberate practice.** The latter two findings were in line with prior expectations namely; the more an individual engages in **deliberate practice** (particularly solitary practice) the more proficient their performance is likely to be. This finding supports one of the main tenets of Ericsson et al.’s (1993) theory whereby expertise is acquired through a vast number of hours spent engaging in activities purely designed to improve performance, i.e., **deliberate practice**.” - Duffy et al. (2004, pp. 242–243). “[I]n a study of district, national, and professional dart players Duffy, Baluch, and Ericsson (2004) found that solitary **deliberate practice** was closely related to performance, whereas the amount of social dart activities did not predict performance.” - Ericsson (2014c, p. 191)
Charness et al. (2005)^2^	“The paper by Charness, Tuffiash, Krampe, Reingold, and Vasyukova [2005]…extends an earlier classic chapter by Charness, Krampe, and Mayr (1996) and examines retrospective estimates by a large sample of chess players about their training during the development of their skill and expertise. This paper reports the most compelling and detailed evidence for how designed training (**deliberate practice**) is the crucial factor in developing expert chess performance.” - Ericsson (2005, p. 237). “In chess, Charness and his colleagues (Charness, Krampe, and Mayr, 1996; Charness, Tuffiash, Krampe, Reingold, and Vasyukova, 2005) have found that the amount of solitary chess study was the best predictor of performance at chess tournaments, and when this type of deliberate practice was statistically controlled, there was no reliable benefit from playing chess games.” - Ericsson (2014c, p. 191)
Tuffiash et al. (2007)^2^	“Several researchers have reported a consistent association between the amount and quality of solitary activities meeting the criteria of **deliberate practice** and performance in different domains of expertise, such as…SCRABBLE (Tuffiash et al., 2007).” - Ericsson et al. (2009, p. 9)
Duckworth et al. (2011)^2^	“Our major findings in this investigation are as follows: **Deliberate practice**—operationally defined in the current investigation as the solitary study of word spellings and origins—was a better predictor of National Spelling Bee performance than either being quizzed by others or engaging in leisure reading. With each year of additional preparation, spellers devoted an increasing proportion of their preparation time to **deliberate practice**…Grittier spellers engaged in **deliberate practice** more so than their less gritty counterparts, and hours of **deliberate practice** fully mediated the prospective association between grit and spelling performance.” - Duckworth et al. (2011, p. 178)

*In each quotation, the boldface emphasis on “deliberate practice” is added. ^1^Rejected because article “do[es] not record assigned individualized practice tasks with immediate feedback and goals for practice” (see Ericsson, 2014b; Table 3). ^2^Rejected because article “do[es] not record a teacher or coach supervising and guiding all or most of the practice” (see Ericsson, 2014b; Table 2). Table from Hambrick et al. (2018b); used with permission from John Wiley and Sons.*

Ericsson’s (2014a) rejection of his own study of darts (Duffy et al., 2004) and his rejection of Charness and colleagues’ studies of chess (Charness et al., 2005) were especially noteworthy (see the Appendix for Ericsson’s varying characterizations of the Charness studies). Ericsson’s (2014a) stated reason for rejecting these studies was that they provided “no record of a teacher/coach supervising all or most of practice” (see Ericsson, 2014b, Table 2). However, in a chapter published in the very same year, Ericsson (2014c) used both these studies to argue for the importance of deliberate practice, stating:

**TABLE 2:** Ericsson’s evaluations of the 14 studies and associated effect sizes included in Ericsson and Harwell’s (2019) meta-analysis, from prior to 2014 to the present.

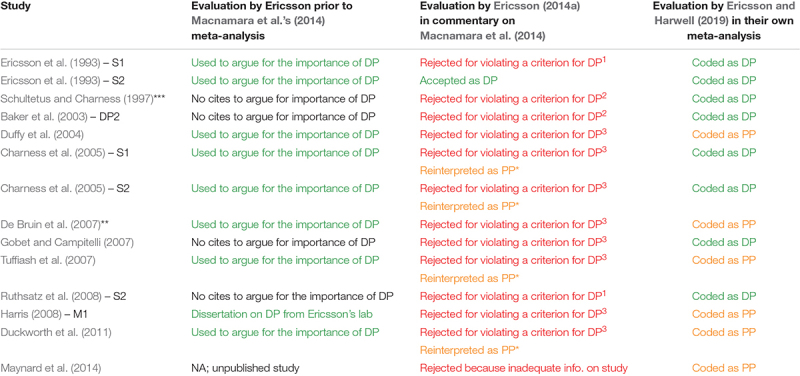

DP, deliberate practice; PP, purposeful practice. Each study/effect size in red was rejected by Ericsson (2014a) for violating one of his criteria at that time for deliberate practice (see Supplementary Tables in Ericsson, 2014b): ^1^“Restriction of range in attained performance and accumulated deliberate practice” (Ericsson, 2014c; Table 5). ^2^“Articles do not record assigned individualized practice tasks with immediate feedback and goals for practice” (Ericsson, 2014c; Table 3). ^3^“Articles do not record a teacher or coach supervising and guiding all or most of the practice” (Ericsson, 2014b; Table 2). *See Moxley et al. (2019), for reinterpretation of studies as focusing on purposeful practice rather than deliberate practice. **Ericsson and colleagues (see Ericsson and Towne, 2013) cite another report of this chess study by de Bruin and colleagues to argue for the importance of deliberate practice (De Bruin et al., 2008), but that article is based on the same data as reported in the De Bruin et al. (2007) article. ***As cited in Deakin and Cobley (2003).

[I]n a study of district, national, and professional dart players Duffy, Baluch, and Ericsson (2004) found that solitary deliberate practice was closely related to performance, whereas the amount of social dart activities did not predict performance (Ericsson, 2014c, p. 191).

In chess, Charness and his colleagues (Charness, Krampe, and Mayr, 1996; Charness, Tuffiash, Krampe, Reingold, and Vasyukova, 2005) have found that the amount of solitary chess study was the best predictor of performance at chess tournaments, and when this type of deliberate practice was statistically controlled, there was no reliable benefit from playing chess games (Ericsson, 2014c, p. 191)^2^.

Another inconsistency was Ericsson’s (2014a) rejection of his own study of spelling bee contestants (Duckworth et al., 2011) for violating this same teacher/coach criterion. Just 2 years earlier, in criticizing a journalist for his description of the study, Ericsson (2012) emphatically stated that the study *had* collected data on deliberate practice:

In that study we (as I was also one of the co-authors) collected data on ‘deliberate practice.’ We found that ‘Grittier spellers engaged in deliberate practice more so than their less gritty counterparts, and hours of deliberate practice fully mediated the prospective association between grit and spelling performance’ (p. 6).

In a commentary on a subsequent meta-analysis of deliberate practice in sports performance, Ericsson (2016) again insisted that our broad definition of deliberate practice was incorrect (for a reply, see Macnamara et al., 2016a). Yet he did not resolve or acknowledge the material inconsistencies in his past descriptions of deliberate practice, especially those concerning the important question of who designs deliberate practice activities (see Figure 2). Furthermore, Ericsson again criticized our inclusion of studies that he had previously used to argue for the importance of deliberate practice (e.g., Helsen et al., 1998; Hodges and Starkes, 1996). It is difficult, if not impossible, for scientists to test a theory if the definition of and standard for evidence are changed repeatedly, with no acknowledgment of and no explanation for the changes (Ferguson and Heene, 2013).

### New Types of Practice

Around this same time, in their aforementioned popular press book *Peak: Secrets from the New Science of Expertise*, Ericsson and Pool (2016) proposed a distinction between deliberate practice and two new forms of practice, perhaps in an attempt to address discrepancies and confusion surrounding the definition of deliberate practice that were being documented in the scientific literature (e.g., Hambrick et al., 2014a). They introduced and defined *naïve practice* as “essentially just doing something repeatedly, and expecting that the repetition alone will improve one’s performance” (p. 14), and *purposeful practice* as an activity that has well-defined, specific goals and involves feedback, but which is self-directed rather than teacher-directed. Ericsson and Pool (2016) explained that “deliberate practice requires a teacher who can provide activities designed to help a student improve his or her performance…. With this definition we are drawing a clear distinction between purposeful practice—in which a person tries very hard to push himself or herself to improve—and practice that is both purposeful *and* informed” (p. 98, emphasis added). They further explained that “some approaches to training are more effective than others” (p. 85) and that deliberate practice is “the most effective method of all…. The gold standard, the ideal to which anyone learning a skill should aspire” (Ericsson and Pool, 2016, p. 85).

Using this new framework, Ericsson and colleagues reinterpreted studies they had once used to argue for the importance of “deliberate practice” as studies of the less effective “purposeful practice,” but without explicitly acknowledging and justifying the reinterpretation (see Macnamara and Hambrick, 2020). As an example, Ericsson (2005) described Charness et al.’s (2005) chess study (entitled *The Role of Deliberate Practice in Chess Expertise*) as providing “the most compelling and detailed evidence for how designed training (deliberate practice) is the crucial factor in developing expert chess performance” (p. 237). Nevertheless, in their recent article, Moxley et al. (2019) explained that “Charness et al. (2005) found evidence for an independent effect of engagement in *purposeful practice* for chess skill” (p. 1163, emphasis added). As another example, Duckworth, Ericsson, and colleagues’ spelling bee study (Duckworth et al., 2011) focused on deliberate practice: The article reporting the study was titled *Deliberate Practice Spells Success: Why Grittier Competitors Triumph at the National Spelling Bee* and the major conclusion of the study was that “[d]eliberate practice mediated the prediction of final performance by the personality trait of grit” (p. 174). Yet the recent Moxley et al. (2019) article stated that “[a]fter the questionnaire, we asked participants to fill out several additional personality measures that Duckworth et al. (2011) had found to be related to *purposeful practice* in preparation for competitions in spelling” (p. 1158, emphasis added).

In another instance, Ericsson and colleagues went from arguing that activities exist that meet the criteria for deliberate practice in the boardgame SCRABBLE, to arguing that it is not possible to engage in deliberate practice in SCRABBLE. Specifically, referring to Tuffiash et al.’s (2007) SCRABBLE study, Ericsson et al. (2009) stated that “[s]everal researchers have reported a consistent association between the amount and quality of solitary activities meeting the criteria of deliberate practice and performance in different domains of expertise, such as…Scrabble (Tuffiash et al., 2007)” (p. 9). However, Moxley et al. (2019) wrote that because SCRABBLE lacks professional coaches “SCRABBLE players cannot engage in deliberate practice, but only purposeful practice and other types of practice” (p. 1150). Under this new framework, activities that once qualified as deliberate practice are now classified as less effective purposeful practice. Of course, it is appropriate for a theorist to reinterpret past evidence as a theory is refined and revised over time. But it is a serious problem, as in this case, when the reinterpretations of evidence are not *explicitly* acknowledged, explained, and justified. In the absence of such transparency, the reader will be led to think that the empirical support for the original theory was stronger than it is or ever was.

### Recent Developments

Two reanalyses of our first meta-analysis (Macnamara et al., 2014) have been published in the past two years. The first was by Miller et al. (2018), who argued that some of the studies that we included in our meta-analysis did not capture deliberate practice. Reanalyzing our data, Miller et al. (2018) explained that they had raters code each study “using only the methods section” (p. 6) and explained that “a study was coded as DP (deliberate practice) if and only if it explicitly indicated it estimated the effects of deliberate practice” (p. 6). Their meta-analysis revealed an average correlation of *r* = 0.40 for “deliberate practice” (compared to our value of *r* = 0.38), and an average correlation of *r* = 0.21 for what they deemed “non-deliberate practice.” However, as we pointed out in a reply (Hambrick and Macnamara, 2019a; see also Hambrick and Macnamara, in press), numerous studies Miller et al. (2018) coded as deliberate practice did not meet their own inclusion criteria. For example, Miller et al. (2018) coded Bilalić et al.’s (2007) chess study as a deliberate practice study. Yet Bilalić et al. (2007) made no mention of “deliberate practice” anywhere in their methods, and elsewhere in their article Bilalić et al. (see pp. 467–468) explicitly stated that they did *not* interpret their practice measures as deliberate practice. This study and numerous others fulfilled the broad definition of deliberate practice we used in our own meta-analysis, but they did not meet Miller et al.’s (2018) narrower definition. There are two possibilities here: Miller et al. (2018) made errors in coding their studies, or they used coding criteria different from those stated in their article.

In a recent reply, while not addressing the coding issue, Miller et al. (2019) highlighted that the average deliberate practice-performance correlation in their reanalysis and in our meta-analysis were very similar, which is an accurate observation (*r* = 0.40 vs. *r* = 0.38). Then they noted, “Still, something about our analyses [Miller et al.’s 2018, reanalysis] was displeasing to [Hambrick and Macnamara, 2019a]” (Miller et al., 2019, p. 289). We were actually clear about what the problem was: Miller et al.’s (2018) meta-analysis included studies that clearly did not meet their own stated inclusion criterion, rendering their results uninterpretable. Miller et al. (2019) added, “The central question of both studies was the role played by deliberate practice in the acquisition of expertize [*sic*]. One might think there would be a ‘meeting of the minds’ when the estimates from their analysis and ours returned such similar results” (p. 289). Miller et al. (2019) seem to suggest here that because their reanalysis yielded an average deliberate practice-performance correlation very similar to the corresponding correlation in our meta-analysis, we should have found their results acceptable. However, findings from scientific research should be evaluated based on whether they are accurate and interpretable, not on whether they agree with findings from one’s own research. On that note, we reiterate that until Miller and colleagues can clarify their methods the results of their reanalysis will only add to the confusion surrounding deliberate practice. As it stands, the results of their reanalysis remain uninterpretable.

The second reanalysis of our dataset was by Ericsson and Harwell (2019). Again, without resolving past inconsistencies in descriptions of deliberate practice in Ericsson and colleagues’ writings, Ericsson and Harwell (2019) criticized our use of a general definition of deliberate practice, stating:

There is no disagreement that the goal of improving performance is one characteristic of deliberate practice, and Ericsson et al. (1993) even wrote that “deliberate practice is a highly structured activity, the explicit goal of which is to improve performance” (p. 368). This sentence was, however, not a definition of deliberate practice any more than the true statement that “a dog is an animal” would imply the inference that “all animals are dogs.” (p. 5).

According to this statement, Ericsson et al. (1993) never proposed the general definition of deliberate practice that we used for our meta-analysis. Yet, Lehmann and Ericsson (1997) stated that “Ericsson et al. (1993) *have defined deliberate practice* as a structured activity designed to improve performance” (p. 47, emphasis added). So, to be clear, Lehmann and Ericsson (1997) stated that Ericsson et al. (1993) defined deliberate practice as a structured activity designed to improve performance, but then Ericsson and Harwell (2019) stated that Ericsson et al. (1993) did *not* define deliberate practice as such. Contradictory statements such as these lead to confusion about what the definition of deliberate practice is—and even whether there is a fixed definition at all.

Compounding the issues already discussed, Ericsson and colleagues’ standard for evidence concerning deliberate practice has apparently shifted yet again. For their main analysis, Ericsson and Harwell (2019) retained 14 of the 88 studies included in our meta-analysis (Macnamara et al., 2014), coding from these studies eight effect sizes as deliberate practice and six effect sizes as purposeful practice^3^. However, as shown in Table 2, seven of the eight effect sizes they coded as deliberate practice were from studies previously rejected by Ericsson (2014a) in his commentary on Macnamara et al.’s meta-analysis for not meeting the criteria for deliberate practice (recall that he rejected 87 of the 88 studies in that commentary). The major reason for this discrepancy is that Ericsson and Harwell (2019) used a more lenient teacher/coach criterion for deliberate practice in their own meta-analysis than Ericsson (2014a) used when evaluating our meta-analysis. More specifically, whereas Ericsson (2014a) required that a study “record a teacher or coach supervising and guiding all or most of the practice” (see Ericsson, 2014b, Table 2), Ericsson and Harwell (2019) required only that a study *describe* individualized sessions with a coach or teacher, with no specification of the lower limit on the amount of supervision and guidance. As they explained:

For coding purposeful versus deliberate practice, we looked for explicit mentions within the original study methods of individualized sessions with coaches or teachers as being included as part of the estimate of solitary practice. If a study did describe individualized instruction sessions as being part of the practice estimate it was considered to be deliberate practice (Ericsson and Harwell, 2019, Supplementary Material, p. 5).

Ericsson and Harwell (2019) do not explain why they made this shift from the stricter criterion that Ericsson (2014a) used when evaluating our meta-analysis to a more lenient teacher/coach criterion for themselves when they reanalyzed our meta-analytic data. Interestingly, they do not even cite Ericsson’s (2014a) commentary, although Ericsson (2018) very recently used it as a basis for rejecting the conclusions of the Macnamara et al. (2014) meta-analysis (see also Ericsson and Pool, 2016).

Additional examples of apparent shifts in the standard for evidence concerning deliberate practice can be found in Ericsson and Harwell (2019). For example, in his earlier commentary on Macnamara et al. (2014), Ericsson (2014a) rejected Ruthsatz et al.’s (2008) studies of musicians as a valid study of deliberate practice because there was a restriction of range in the measures (see Ericsson, 2014b, Table 5). However, Ericsson and Harwell (2019) apparently no longer saw this as a problem, because they coded one of these previously rejected studies (Study 2) as deliberate practice and included it in their own meta-analysis.

Setting aside these issues, what would the results of Ericsson and Harwell’s (2019) meta-analysis indicate if they were taken at face value? After their correction for measurement error variance (unreliability), the average correlation for the 14 studies that measured either purposeful practice or deliberate practice with performance increased from *r* = 0.54 to *r* = 0.78, indicating that purposeful/deliberate practice^4^ explained 61% of the reliable variance in the performance. Although psychometric corrections come with larger standard errors and confidence intervals (Oswald et al., 2015), this point estimate of 61% might be interpreted to support Ericsson et al.’s (1993) central claim that individual differences in domain-relevant performance can largely be accounted for by accumulated amount of practice—at least a weak version of this claim (see Hambrick et al., 2018a).

However, it is critical to examine Ericsson and Harwell’s (2019) assumptions about the reliability of the variables, because those values determined the degree of correction to the correlation (i.e., the lower the assumed reliability, the greater the correction). As previously noted, Ericsson and colleagues previously stated that “self-report practice estimates repeatedly from experts in sports and music have reported test-retest reliabilities at or above 0.80” (Tuffiash et al., 2007, p. 129) and that “[t]he collected reliability of cumulated life-time practice at different test occasions in large samples has typically been found to range between 0.7 and 0.8” (Ericsson, 2012, p. 534). However, for reasons that are unclear, Ericsson and Harwell (2019) used a lower reliability estimate of 0.60 for purposeful/deliberate practice, apparently drawing on different information about the reliability of practice. This raises the important question of whether, by using a lower reliability value, they overcorrected the correlation between purposeful/deliberate practice and performance, and thus overestimated the variance shared between the two variables.

As is the case for most measured variables in psychological research, the reality is that the reliability of practice and performance variables must be estimated and can never be known with certainty; the accuracy of any reliability estimate will vary with the number of participants, the number of items/questions on the instrument, and other factors. Furthermore, the reliability of practice and performance variables may vary depending on factors such as the domain, the skill level and age of the participants, and so on. Thus, an approach we have adopted in our own research is to correct correlations between these variables, based on whatever point estimates of reliability are available, but also to report a sensitivity analysis in which the correlation is corrected under both lower and higher levels of reliability (e.g., Macnamara et al., 2014, Table S1).

Table 3 presents such a sensitivity analysis for the meta-analytic correlations that Ericsson and Harwell (2019) report between purposeful/deliberate practice and performance (*r* = 0.54, *k* = 14), between purposeful practice and performance (*r* = 0.51, *k* = 6), and between deliberate practice and performance (*r* = 0.56, *k* = 8). As can be seen, the strength of the correlations varies depending on the reliability estimates, with larger corrections for lower reliability estimates. For example, if reliability is assumed to be *r*_*xx*_ = 0.80 for deliberate practice (Ericsson, 2013) and *r*_*yy*_ = 0.80 for performance, then the corrected correlation of performance with deliberate practice is *r*_*c*_ = 0.70, indicating that deliberate practice explains 49% of the reliable between-person variance in performance (rather than 61%). Thus, conclusions will vary considerably depending on what values are used as the reliability estimates in the psychometric correction.

**TABLE 3:** Sensitivity analyses for Ericsson and Harwell (2019) meta-analytic correlations.

	Performance *r*_*yy*_			
	
Purposeful/Deliberate *r*_*xx*_	0.60	0.70	0.80	0.90
0.60	0.90 (81.0%)	0.83 (69.4%)	**0.78 (60.8%)**	0.73 (54.0%)
0.70	0.83 (69.4%)	0.77 (59.5%)	0.72 (52.1%)	0.68 (46.3%)
0.80	0.78 (60.8%)	0.72 (52.1%)	0.68 (45.6%)	0.64 (40.5%)
0.90	0.73 (54.0%)	0.68 (46.3%)	0.64 (40.5%)	0.60 (36.0%)

	**Performance *r*_*yy*_**			
	
**Purposeful *r*_*xx*_**	**0.60**	**0.70**	**0.80**	**0.90**

0.60	0.85 (72.3%)	0.79 (61.9%)	**0.74 (54.2%)**	0.69 (48.2%)
0.70	0.79 (61.9%)	0.73 (53.1%)	0.68 (46.4%)	0.64 (41.3%)
0.80	0.74 (54.2%)	0.68 (46.4%)	0.64 (40.6%)	0.60 (36.1%)
0.90	0.69 (48.2%)	0.64 (41.3%)	0.60 (36.1%)	0.57 (32.1%)

	**Performance *r*_*yy*_**			
	
**Deliberate *r*_*xx*_**	**0.60**	**0.70**	**0.80**	**0.90**

0.60	0.93 (87.1%)	0.86 (74.7%)	**0.81 (65.3%)**	0.76 (58.1%)
0.70	0.86 (74.7%)	0.80 (64.0%)	0.75 (56.0%)	0.71 (49.8%)
0.80	0.81 (65.3%)	0.75 (56.0%)	0.70 (49.0%)	0.66 (43.6%)
0.90	0.76 (58.1%)	0.71 (49.8%)	0.66 (43.6%)	0.62 (38.7%)

*The table provides corrected correlation coefficients (*r*_*c*_s) based on Ericsson and Harwell’s (2019) reported meta-analytic correlations of *r*_*purposeful/deliberate*_ = 0.54, *r*_*purposeful*_ = 0.51, and *r*_*deliberate*_ = 0.56. Values in parentheses are variance estimates (i.e., *r*_*c*_^2^ × 100). *r*_*xx*_, reliability estimate for practice; *r*_*yy*_, reliability estimate for performance; DP, deliberate practice. The bolded values are the corrected correlations using Ericsson and Harwell’s (2019) reliability values. The formula for computing a correlation corrected for unreliability (*r*_*c*_) is the observed correlation (*r*_*obs*_) divided by the square root of the product of the variables’ reliabilities (*r*_*xx*_ and *r*_*yy*_): *r*_*c*_ = *r*_*obs*_/√(*r*_*xx*_ × *r*_*yy*_) (see Schmidt and Hunter, 1999).*

Across the board, the correlations between practice and domain-relevant performance (expertise) in Table 3 are meaningfully large, from theoretical, statistical, and practical perspectives. At the same time, the correlations vary considerably in magnitude and may lead to different conclusions about the importance of the practice variables. We illustrate this point with reference to the deliberate practice correlations (the bottom set of results in Table 3). If deliberate practice explained, for example, 87% of the variance in performance (assuming relatively low reliabilities of *r*_*xx*_ = 0.60 and *r*_*yy*_ = 0.60), then a strong version of Ericsson et al.’s (1993) claim that individual differences in performance can largely be accounted for by deliberate practice would be supported. However, if deliberate practice explained just over half (56%) of the variance (assuming higher reliabilities of *r*_*xx*_ = 0.80 and *r*_*yy*_ = 0.70), then a weaker version of the claim would be supported. If deliberate practice explained an even smaller amount of the variance—for example, 39% (assuming even higher reliabilities of *r*_*xx*_ = 0.90 and *r*_*yy*_ = 0.90)—then an implication would be that factors other than deliberate practice explain more of the variance in performance than deliberate practice does. These different variance estimates might then lead to different priorities in expertise research. For example, the first scenario (87% of variance explained) might prompt an exclusive focus on training history, whereas the third scenario (39% of variance explained) might prompt a broader focus on multiple determinants of performance differences (e.g., training history, basic abilities). Elsewhere, we have argued that based on extant evidence, research should indeed investigate the role of a wide range of factors in explaining individual differences in expertise (Ullén et al., 2016; Hambrick et al., 2018b).

We further note that, if they were taken at face value, Ericsson and Harwell’s (2019) findings would fail to support a central prediction of the latest version of the deliberate practice view. As mentioned earlier, Ericsson and Pool (2016) differentiated deliberate practice from naïve practice and purposeful practice, describing deliberate practice as “the most effective method of all…the gold standard” (p. 85). A straightforward prediction, following from these claims, is that the positive correlation between deliberate practice and domain-relevant performance should be significantly greater than the positive correlation between purposeful practice and domain-relevant performance (i.e., *r*_*deliberate*_ > *r*_*purposeful*_). Ericsson and Harwell’s (2019) findings do not support this prediction: the average correlation was *r* = 0.56 for deliberate practice and *r* = 0.51 for purposeful practice, a non-significant difference (*p* = 0.64). Ericsson and Harwell (2019) report this finding, but they note only that “practice was positively associated with performance whether it was conducted under the guidance of a coach or teacher” (p. 11). From the standpoint of the distinction between the two types of practice (Ericsson and Pool, 2016), the equally important conclusion would seem to be that there is no evidence from the meta-analysis that deliberate practice has higher validity than purposeful practice in predicting individual differences in domain-relevant performance, as is predicted by Ericsson and colleagues’ new framework.

## How Important Is Deliberate Practice?

Again, how important is deliberate practice as a predictor of individual differences in expertise? It is somewhat difficult to say given the ambiguity over the definition of deliberate practice, but we can at least summarize evidence from meta-analyses by different groups of researchers that have attempted to answer this question. Along with the aforementioned reanalysis of chess and music studies (Hambrick et al., 2014b), there have been five formal meta-analyses of the deliberate practice-performance relationship. Table 4 summarizes the overall result from these meta-analyses (i.e., the overall correlation between deliberate practice and performance), and presents a sensitivity analysis showing variance estimates under different reliability assumptions, from unacceptable to excellent. As shown, across meta-analyses, deliberate practice explains a sizeable amount of the between-person variance in performance. However, we conclude that it is unlikely to be as important as Ericsson and colleagues have hypothesized it is. In nearly all cases, deliberate practice leaves a large amount of reliable variance unexplained, and in most cases, the unexplained variance exceeds the explained variance. Our conclusion, as in the past, is that deliberate practice is an important predictor of individual differences in expertise. However, deliberate practice is it is unlikely to be as important as Ericsson and colleagues have proposed it is.

**TABLE 4:** Results of meta-analyses of deliberate practice–performance relationship.

Study	Domain	*K*	*r* (95% CI)	% variance	<Acceptable (*r*_*xx*_, *r*_*yy*_ = 0.60)	Acceptable (*r*_*xx*_, *r*_*yy*_ = 0.70)	Good (*r*_*xx*_, *r*_*yy*_ = 0.80)	Excellent (*r*_*xx*_, *r*_*yy*_ = 0.90)
Macnamara et al. (2014)	Multiple	157	0.38 (0.33, 0.42)	14.4%	0.63 (40.1%)	0.54 (29.5%)	0.48 (22.6%)	0.42 (17.8%)
Platz et al. (2014)	Music	14	0.61 (0.54, 0.67)	37.2%	1.00 (100%)	0.87 (75.9%)	0.76 (58.1%)	0.68 (45.9%)
Macnamara et al. (2016b)	Sports	63	0.45 (0.36, 0.53)	20.3%	0.75 (56.3%)	0.64 (41.3%)	0.56 (31.6%)	0.50 (25%)
Miller et al. (2019)	Multiple	70	0.40 (0.34, 0.46)	16.0%	0.67 (44.4%)	0.57 (32.7%)	0.50 (25%)	0.44 (19.8%)
Ericsson and Harwell (2019)	Multiple	8	0.56 NR	31.4%	0.93 (87.1%)	0.80 (64.0%)	0.70 (49%)	0.62 (38.7%)

**k*, number of effect sizes. *r*, meta-analytic correlation between deliberate practice and performance.% variance, percentage of variance in performance explained by deliberate practice (i.e., *r*^2^). For the corrected correlations, values in parentheses adjacent to correlations are variance estimates (*r*_*c*_^2^ × 100). NR, not reported.*

## Is the Deliberate Practice View Defensible?

In view of the issues discussed in the preceding pages, it seems reasonable to ask whether the deliberate practice view is scientifically defensible—that is, whether deliberate practice can be conceptualized and tested empirically in a consistent manner by the research community. In his book *The Logic of Scientific Discovery*, the philosopher of science Karl Popper (1959) argued, “In so far as a scientific statement speaks about reality, it must be falsifiable; and in so far as it is not falsifiable, it does not speak about reality” (p. 314). By definition, a theory is unfalsifiable when it cannot be rejected under any circumstances, because it can accommodate any finding. This happens when multiple, contradictory definitions of theoretical concepts are proposed by a theorist, and when the theoretical and operational criteria are kept in a fluid state. Under these conditions, evidence can be rejected or accepted depending on whether it supports the theory.

When a theory becomes unfalsifiable, it ceases to be a *scientific* theory, at least in the Popperian sense. The theory is always “right” and cannot be evaluated against competing theories. Ferguson and Heene (2013) described a theory that has entered this state as being “undead,” like a zombie that is technically dead but remains animate. The undead theory “continues in use, having resisted attempts at falsification, ignored disconfirmatory data, negated failed replications through the dubious use of meta-analysis or having simply maintained itself in a fluid state with shifting implicit assumptions such that falsification is not possible” (Ferguson and Heene, 2013, p. 559).

Is the deliberate practice view falsifiable? We will leave it to readers of this article to draw their own conclusions. At the very least, it seems difficult to deny that there are serious problems with the deliberate practice view, as Ericsson and colleagues have presented it over the past 25 years. As we have documented here, Ericsson and colleagues have described deliberate practice in contradictory ways, creating major confusion about the definition of deliberate practice. Furthermore, Ericsson and colleagues’ standard for evidence—the specific criteria that need to be satisfied to use a study to argue for the importance of deliberate practice and even the criteria themselves—has appeared to shift multiple times.

To be sure, it is not only normal, but expected, for a scientist to revise a theory as evidence relevant to that theory accumulates through research. Theory revision is a fundamental part of what the philosopher of science Imre Lakatos (1978) called a “progressive” program of research. In this iterative process, revisions are explicitly acknowledged and clearly explained and justified so that they can be understood and critically evaluated by other scientists. When theory revisions are *not* made in a transparent manner, then in Lakatos’ terminology a theory can be endlessly adjusted and readjusted to keep it “alive.” The program of research then shifts from “progressive” to “degenerative” (see also Musgrave and Pigden, 2016).

On a related note, the concept of deliberate practice is arguably underspecified in ways that leave open the opportunity for numerous *post hoc* explanations of results. For example, Ericsson and colleagues have stated that deliberate practice requires that the performer have “full concentration” (e.g., Ericsson and Harwell, 2019). However, this is a psychological state that may be impossible to achieve. Would, for example, a person’s awareness of the environment (e.g., the temperature) or a fleeting thought (e.g., about an event earlier in the day) mean that they were not fully concentrating on the training task? If yes, then it seems unlikely that a person could ever fulfill this criterion of deliberate practice. Furthermore, we are not aware of a method for objectively determining whether a person has *full* concentration on something. Research approaches from cognitive psychology (e.g., primary-secondary task paradigms) permit no more than relative statements about the degree to which a person is attending to one task (or stimulus) versus some other task(s). Except for subjective self-reports, we are also unaware of attempts by Ericsson and colleagues themselves to measure concentration level during practice. If a criterion for a theoretical construct (e.g., achieving “full concentration”) either cannot be achieved or cannot be empirically verified, then imposing that criterion makes the theory unfalsifiable. This sort of flexibility in the deliberate practice view, along with the definitional confusion we have discussed, presents an additional problem for its scientific viability.

Why is it important for researchers to comment publicly when a theory in their research area appears to be degenerating? It is important because a degenerating theory—especially if it is influential—impedes actual progress in an area of research. The value of research to test the theory becomes questionable, because evidence is accepted as valid only if it supports the theory and rejected if it fails to support the theory. In turn, practical recommendations and applications based on the theory will lack a scientific foundation, because even conflicting recommendations and applications can be supported. If conclusions from this research do not have a solid empirical foundation, then recommendations based on the theory may be wasteful, counterproductive, or even harmful.

To be certain, we do not think that the concept of deliberate practice should be abandoned. Deliberate practice is a vital area of research in psychological science and other fields. However, given the issues we have discussed at length in this article, we do believe that it would be highly beneficial to expertise researchers (and scientists from other research areas interested in expertise) for proponents of the deliberate practice view to fully address and resolve the apparent inconsistencies and shifts in the definition of and standard of evidence for deliberate practice that we have documented here—and which raise serious concerns about the viability of the deliberate practice view as a scientific theory. This would allow other researchers to empirically evaluate the importance of deliberate practice as a predictor of individual differences in expertise, both in individual studies and meta-analyses, and to compare its predictive validity to that of other factors (e.g., general aptitudes, basic capacities) and the conditions of practice (e.g., spacing of practice sessions, type of feedback).

To this point, in the following sections, we summarize key evidence for the role of a diverse range of factors in explaining individual differences in expertise, and then discuss in broad terms what we believe are fruitful directions for future research to develop comprehensive models of expertise.

## Toward a Multifactorial Model of Expertise

What might explain individual differences in expertise, beyond any contribution of deliberate practice? We direct readers to recent theoretical/review articles in which we discuss this issue at length (Hambrick et al., 2016; Ullén et al., 2016; Macnamara and Hambrick, 2020). Here, we briefly summarize evidence concerning three major classes of factors.

### Developmental Factors

The question of when specialized training should commence in a person’s life is the subject of a longstanding debate in the field of expertise. The *early specialization view* argues that the earlier the training can begin, the better. The logic of this view is straightforward: Because it is both physically and psychologically taxing, a person can engage in only a few hours of deliberate practice a day (around 4 h on average; Ericsson et al., 1993) without burnout and/or injury. Therefore, the individual who begins training at a relatively late age (e.g., age 12) can never catch up to the individual who begins training earlier (e.g., age 6). However, in a meta-analysis of sports studies with samples representing a wide range of skill, we found no evidence for an earlier average starting age for high-skill athletes relative to lower-skill athletes. Furthermore, research suggests that the highest (elite) levels of sports performance are associated with a later starting age, combined with participation in a diverse range of sports in adolescence. For example, Güllich (2017) compared 83 international medalists (Olympic/World championship) to 83 non-medalists matched on sport, age, and gender. Up to age 18, the medalists had, on average, accumulated significantly *fewer* hours of organized training/practice in their main sport (by 948 h) than the non-medalists. Moreover, the average starting age was *later* by approximately a year-and-a-half for the medalists compared to the non-medalists. One possible explanation for this finding is that a starting age that is too early increases the risk for injury and/or burnout. Another possible explanation is that starting later allows for more early diverse experiences, increasing the likelihood that the individual will find a sport that is a good match to his or her profile of performance-relevant traits (Güllich, 2017).

### Experiential Factors

A central tenet of the deliberate practice view is that deliberate practice is more predictive of individual differences in expertise than other forms of experience, such as work and play. As Boot and Ericsson (2013) explained, “Ericsson and colleagues…make a critical distinction between domain-related activities of work, play, and deliberate practice, and claim that the amount of accumulated time engaged in deliberate practice activities is the primary predictor of exceptional performance” (p. 146). The available evidence does not appear to support this claim. As already mentioned, if Ericsson and Harwell’s (2019) findings are taken at face value, they reveal that deliberate practice, although claimed to be the “gold standard” for improving performance (Ericsson and Pool, 2016), is not a significantly stronger predictor of individual differences in expertise than mere purposeful practice (i.e., *r*_*deliberate*_ = 0.56 vs. *r*_*purposeful*_ = 0.51, difference in *r*s non-significant). Furthermore, measures of deliberate practice have not always been found to be stronger predictors of individual differences in expertise than measures seeming to meet the definition of “work” and “play.” For example, in a study of insurance salespeople, Sonnentag and Kleine (2000) found that correlations between sales performance and measures of deliberate practice (*r*_*current*_ = 0.21 and *r*_*accumulated*_ = 0.13) were not stronger than the correlation between sales performance and a measure fitting the description of work for this domain—the number of cases handled (*r* = 0.37; see Hambrick et al., 2016, for other examples).

Evidence further suggests that diverse forms of experience are important as well, especially in the early stages of training. For example, in Güllich’s (2017) study comparing the 83 international medalists and 83 non-medalists, he not only found that the medalists had accumulated significantly less main-sport practice than their less-accomplished counterparts during childhood/adolescence, but also that the medalists had accumulated significantly more experience with other sports during this period (see also Güllich et al., 2017).

### Ability Factors

Research has firmly established that cognitive ability explains a statistically and practically significant amount of the variability in people’s acquisition of complex skills (Hambrick et al., 2019; also see Ackerman, 1987, for a review of early studies). That is, people higher in cognitive ability learn complex skills more readily and rapidly than people lower in cognitive ability. For example, in a study of music training, participants with little or no experience playing music completed tests of cognitive ability, music aptitude, and growth mindset, and then they were given instruction in playing a simple piece of music on the piano (Burgoyne et al., 2019). Higher-ability participants showed a greater rate of learning than lower-ability participants, with a general intelligence factor explaining approximately 30% of the individual differences in learning rate.

Ericsson (2014d) has theorized that general cognitive ability is important initially in acquiring complex skills, but its predictive power diminishes as domain-specific skills and knowledge are acquired, stating:

For individuals who have acquired cognitive structures that support a high level of performance the expert performance framework predicts that these acquired cognitive structures will directly mediate superior performance and thus diminishing correlations between general cognitive ability and domain-specific performance (p. 84).

For complex tasks of interest to expertise researchers, evidence for this claim, which we termed the *circumvention-of-limits hypothesis* (Hambrick and Meinz, 2011), is weak and inconsistent. In a recent review (Hambrick et al., 2019), we searched through approximately 1,300 articles and identified 15 studies in the domains of games, music, sports, science, medicine/surgery, and aviation relevant to this hypothesis. Of the 15 studies, only three yielded any evidence supportive of the circumvention-of-limits hypothesis. Moreover, methodological limitations (e.g., small *N*s, measures with unknown or unreported reliability) precluded any strong conclusions from those few studies. Providing what might be considered the strongest evidence for the hypothesis, one of these three studies that seem to support the circumvention-of-limits hypothesis was a meta-analysis of chess studies (Burgoyne et al., 2016; see also Burgoyne et al., 2018, corrigendum). As determined by a moderator test, fluid intelligence correlated significantly more strongly with chess rating in lower-skill chess players (avg. *r* = 0.32) than in higher-skill chess players (avg. *r* = 0.14). However, it is important to note that skill level was highly confounded with age (i.e., lower-ability samples were youth, whereas higher-ability samples were adults), limiting the strength of the evidence in support of the circumvention-of-limits hypothesis.

We also note that results that have sometimes been used to argue that the influence of general cognitive ability on expertise diminishes with increasing skill do not warrant this conclusion. For example, Ericsson (2014d) pointed to results by Ruthsatz et al. (2008) as support for this hypothesis. Ruthsatz et al. (2008) found that a measure of general cognitive ability (Raven’s Progressive Matrices score) correlated positively and significantly with musical accomplishment in high school band members (*r* = 0.25, *p* < 0.05), but not in university music majors (*r* = 0.24) or conservatory students (*r* = 0.12). However, the critical question is not whether the lower-skill group correlation is statistically significant while the higher-skill group correlations are not. Rather, it is whether the former correlation and the latter correlations are significantly *different from each other*, as determined by the appropriate statistical test. As it happens, in the Ruthsatz et al. (2008) study, the correlations are not significantly different from each other (all *z* tests for differences in correlations are statistically non-significant). Thus, the results of Ruthsatz et al.’s (2008) study *fail* to support the hypothesis that ability-performance correlations diminish with increasing skill.

**FIGURE 3:**
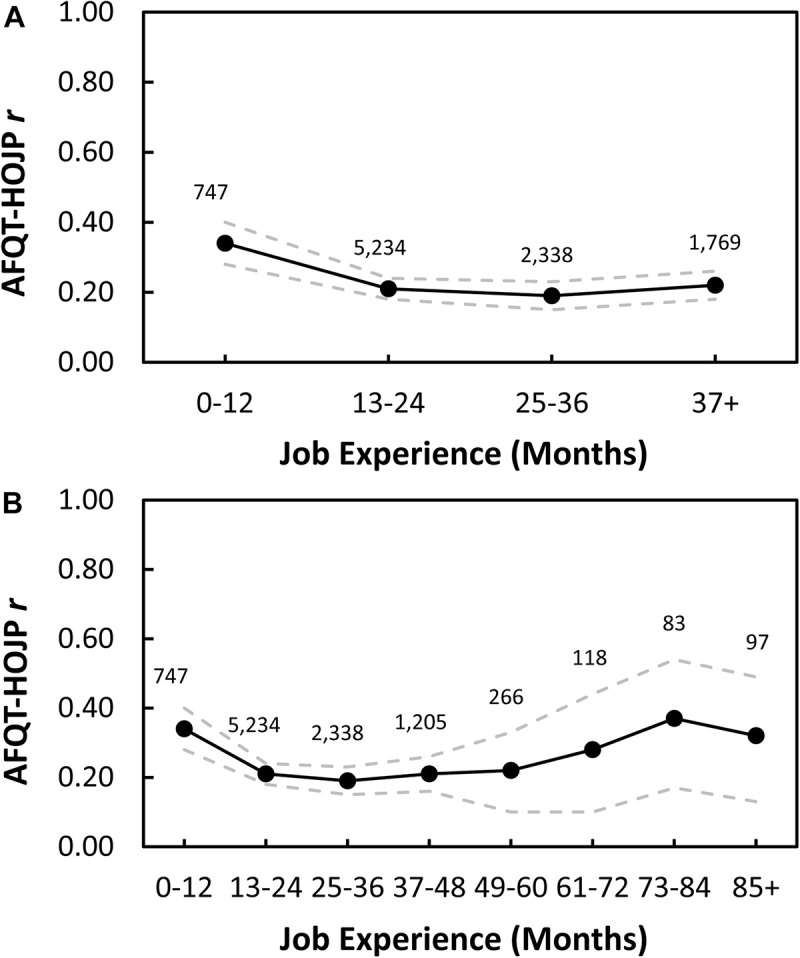
Correlation between the Armed Forces Qualifying Test (AFQT) score and hands-on job performance (HOJP) for 31 military jobs (*N* = 10,088). **(A)** shows AFQT-HOJP correlations when four levels of job experience are created; **(B)** shows this correlation when eight levels of job experience are created. Figure from Hambrick et al. (2019); used with permission of Oxford University Press.

We also reviewed evidence relevant to the circumvention-of-limits hypothesis from the job performance literature, and here the evidence is more consistent and interpretable. General cognitive ability is regarded as the single best predictor of job training performance, and of subsequent job performance (Schmidt and Hunter, 2004; Schmidt, 2014). Higher ability people tend to learn job skills more rapidly and to a higher level than lower ability people, and in turn have greater success on the job. Furthermore, although the validity of general cognitive ability for job performance may drop somewhat as a function of job experience initially, it appears to remain a statistically significant predictor even at high levels of job experience. For example, in a study of 10,088 military personnel across 31 jobs, the correlation between a measure of general cognitive ability (the AFQT score) and hands-on job performance decreased as a function of job experience from 1 to 2 years of job experience (*r* = 0.34 to *r* = 0.21; *z* test for difference = 3.60, *p* < 0.001), but then stabilized and remained statistically and practically significant (see Figure 3). In their own review of the job performance literature, Reeve and Bonaccio (2011) concluded that “although validities might degrade somewhat over long intervals, we found no evidence to suggest that they degrade appreciably, thereby retaining practically useful levels of validity over very long intervals” (p. 269).

### Genetic and Environmental Influences

Research in the field of behavioral genetics has demonstrated that both genetic and environmental variance across individuals contribute to the total variance in a wide range of behavioral outcomes (Turkheimer, 2000), including ability factors that have been found to correlate with measures of expertise. The extent of the genetic contribution is captured by the heritability statistic (*h*^2^), an estimate of the proportion (0 to 1) of the total variance in a trait that can be attributed to genetic (non-environmental) variance within a sample of individuals (Plomin et al., 2008). Because some of these factors correlate with expertise, it stands to reason that both genetic and environmental variance may also contribute to the total variance in expertise. Furthermore, basic abilities and characteristics that may predict individual differences in expertise have also been observed to be substantially heritable, including drawing ability (Arden et al., 2014), music aptitude (Ullén et al., 2014; see Mosing et al., 2018, for a review), and maximal oxygen consumption in athletic performance (VO_2*max*_; Schutte et al., 2016).

At the same time, no psychological trait is 100% heritable (Turkheimer, 2000), and even the most heritable psychological trait will have a sizeable environmental component. For example, heritability estimates for measures of general cognitive ability are typically in the 50 to 70% range in samples drawn from developed countries (e.g., Tucker-Drob and Bates, 2016), with the remaining variance (as much as 50%) explained by shared and/or non-shared environmental factors. This means that correlations between a measure of some trait (e.g., general cognitive ability) and a measure of expertise could be driven by the genetic variance or the environmental variance in the trait measure, or by both types of variances. In other words, the finding that a measure of a heritable trait correlates with expertise is only *consistent with the possibility* that genetic variance is a component of individual differences in expertise.

It is also critical to note that genes and environments cannot generally be assumed to be uncorrelated across people. Rather, across people, genetically influenced factors may contribute to variance in the environments which people seek out and are exposed to. This is the idea of *gene-environment correlation*, or *r*GE (Plomin et al., 1977). For example, just as children who are tall might be more interested in playing basketball and more likely to be selected to play on basketball teams than children who are shorter, those with a high level of music aptitude may be more likely to take up, be selected for, and persist in music than those with a lower level of this aptitude. Consistent with this sort of speculation, there is now evidence to indicate that the propensity to practice in a domain is substantially heritable. In a large twin study, Mosing, Ullén, and colleagues found an average heritability of around 50% for accumulated amount of music practice (Mosing et al., 2014; see also Hambrick and Tucker-Drob, 2015). A possible explanation for this finding is that music aptitude, as well as more general ability and non-ability factors, differentially predispose people to engaging in music practice.

Genetic factors and environmental factors may not only correlate with one another; they may interact in influencing behavioral outcomes—what is known as *gene-environment interaction*, or G × E. G × E occurs when a genetically influenced factor moderates (increases or decreases) the effect of an environmental factor on an outcome. As one example of G × E, analyzing data from the National Merit Twin Study, Hambrick and Tucker-Drob (2015) found that heritability of a music accomplishment variable was 0.43 for individuals who reported engaging in music practice, versus 0.01 for those who did not. (This result was not due to range restriction, as there was still variability in music accomplishment among participants who reported not practicing). This finding suggests that music practice may activate genetic factors that vary across people.

Four additional points concerning the potential contribution of genetic factors to individual differences in expertise are important to note here. First, even if a measure of expertise is found to be heritable, this in *no way* implies that training is unnecessary to develop a high level of expertise, or that training is beneficial to only some people. Training is necessary and essential for developing a high level of expertise in a domain, and except when a condition rules out some type of training (e.g., a visual training regimen for a person who is blind), anyone would be expected to benefit from proper training. Second, heritability does not imply immutability. For example, in adults, height is highly heritable and relatively fixed, whereas weight is similarly heritable but can be modified through an environmental intervention—namely, dieting. Third, environmental interventions that change individual differences will also change heritability. For example, if an environmental intervention were introduced that allowed nearly everyone to reach about the same level of skill in some task, heritability would be expected to decrease. In a similar manner, heritability can differ across populations (e.g., in a developing country vs. a non-developing country; Tucker-Drob and Bates, 2016). Fourth, and finally, it is safe to assume that to the degree that expertise is heritable, this would reflect variation in a great many genetic variants (i.e., single nucleotide polymorphisms, or SNPs), meaning there is no “expertise gene” in any domain. As Chabris and colleagues noted, “A typical human behavioral trait is associated with very many genetic variants, each of which accounts for a very small percentage of the behavioral variability” (Chabris et al., 2015, p. 305). Research is uncovering genetic variants that may contribute to individual differences in expertise, but it is highly unrealistic to expect that any one of these factors will account for a large amount of the variance in expertise.

### Putting It All Together

**FIGURE 4:**
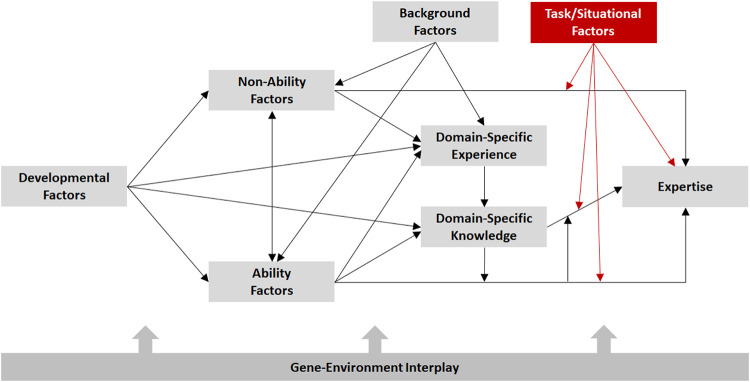
Multifactorial model of expertise. From Hambrick et al. (2018c). Used with permission of Routledge.

Taken together, evidence suggests that individual differences in expertise arise from influences of multiple factors. This includes training and other forms of domain-relevant experience, as well as developmental factors (e.g., age of starting training), ability factors (e.g., aptitudes), non-ability factors (e.g., personality traits), and background factors (e.g., opportunity to engage in training). In recent articles, we have proposed the *multifactorial gene-environment interaction model* (MGIM) to describe how these factors relate to one another and to serve as a guide for future research on expertise (Ullén et al., 2016). As illustrated in Figure 4, the MGIM assumes that expertise arises from influences of both domain-general and domain-specific factors, which are assumed to be influenced by both genetic and environmental factors. The model further assumes that task/situational factors may moderate the influence of these factors on expertise (i.e., domain-relevant performance).

## The Path Ahead in Expertise Research

Ericsson and colleagues’ deliberate practice view has had a monumental impact on the field of expertise, and is important in the history of psychology, more generally. However, in our assessment, it is not clear that the deliberate practice view is defensible as a scientific theory. As described here in some detail, Ericsson and colleagues have defined deliberate practice in inconsistent ways (see Figure 2) and the standard for evidence concerning deliberate practice has appeared to shift multiple times (see Tables 1, 2). These issues present problems for the empirical testability (i.e., falsifiability) of the deliberate practice view.

Embracing tenets of the *open science movement*, we believe that the path ahead is for expertise researchers to work together to develop testable theories that take into account a much wider range of potentially relevant causal constructs than have often been considered in previous research, and to use rigorous empirical methods to evaluate these theories. The open science movement promotes normative values aimed at increasing accuracy, openness, and fairness in scientific research and scholarship (see a special issue of the *Journal of Expertise* devoted to open science in expertise research; McAbee and Macnamara, 2020).

Nearly 80 years ago, the sociologist of science Robert Merton (1942) described four scientific “imperatives” that capture the values of the open science movement. First, *universalism*: scientific validity is independent of the status of the people conducting the research; evidence should be evaluated based on its own merits rather than the status or prominence of the person reporting the evidence. Second, *communism:* a theory does not belong to the theorist, it belongs to the field. The theorist has no greater right to the theory once it is made public than any other scientist. Third, *disinterestedness*: scientists should perform research to increase understanding of some phenomenon rather to advance self-interests, whatever they may be. Finally, *organized skepticism*: a field should scrutinize claims based on empirical evidence.

In the wake of the replication crisis in the social sciences, many measures have been proposed to increase the reproducibility of research findings in psychological science and to accelerate progress in research (see Munafò et al., 2017). Preregistration of study designs, primary outcomes, and data analysis plans can help safeguard against *post hoc* interpretation of data. Improved methodological training can help researchers avoid pitfalls in designing studies (e.g., omitting critical control conditions) and in data analysis (e.g., misinterpreting *p*-values). Collaboration can facilitate collection of large samples and help to ensure that multiple theoretical perspectives are considered in study design. The time is also ripe for a preregistered “adversarial collaboration”—a study in which researchers with differing views agree on an empirical test to resolve a theoretical dispute that is designed to provide a fair test of both views (see Kahneman and Klein, 2009; for a recent example, see Doherty et al., 2019). No less so than in any area of psychological research, we believe that open science will accelerate progress toward greater understanding of the nature and origins of expertise and expert performance.

### Recommendations for Expertise Research

We make four general recommendations for conducting expertise research, which are based on best research practices in differential psychology (see Ackerman and Hambrick, 2020). First, after selecting a domain for the research, the researchers should seek to assess a wide range of potentially relevant causal factors (Simonton, 1999). Whatever the domain, it will be critical to measure key environmental factors, including various types of training and factors related to the opportunity to engage in these activities. However, drawing on the vast literature in differential psychology, it will be equally critical to include measures of basic abilities, capacities, dispositions, and other psychological traits that may affect performance directly, or indirectly through training. Only then can the relative and joint contributions of these factors to individual differences in expertise be evaluated.

The second recommendation is that multiple measures be used to index each of the hypothesized constructs. It is axiomatic in the psychological methods literature that virtually no observed measure (or indicator) is “construct pure.” That is, a score collected by an instrument (test, questionnaire, etc.) designed to measure a given hypothetical construct may reflect that construct to some degree, but it will certainly reflect other, construct-irrelevant factors, such as participants’ familiarity with a particular method of assessment (e.g., test format) and psychological states that may affect their responding (e.g., sleep deprivation and motivation). There is no perfect way to deal with this problem, but when multiple measures of a construct are obtained, it becomes possible to use data-analytic techniques (viz., structural equation modeling) that are explicitly designed to deal with this issue by allowing researchers to model latent variables that are closer to theoretical constructs of interest than observed variables are.

The third recommendation is that the sample of participants from the targeted domain should ideally represent a wide range of performance rather than extreme groups. As we have noted elsewhere (Hambrick et al., 2019), categories such as “novice” and “expert” are not naturally occurring—they are groups of performers created based on ultimately arbitrary cuts on performance scores. Accordingly, scientific research on expertise should endeavor to explain the full range of performance differences within different domains rather than differences between artificial groups of performers, and also continuities and discontinuities across this range (see Bliese and Lang, 2016).

The final recommendation is for expertise researchers to begin large-scale longitudinal studies. Longitudinal studies are expensive and, by their very nature, time-consuming. At the same time, they are common in psychology. For example, there are longitudinal studies in the area of cognitive aging that have been running for many decades, such as the Seattle Longitudinal Study (Schaie, 2005). There is also precedent for longitudinal studies of expertise, including Schneider and colleagues’ important longitudinal study of tennis skill, which included two future World No. 1 players (Schneider et al., 1993). Although expensive, labor-intensive, and time-consuming, multi-site longitudinal studies of expertise will provide for much stronger conclusions concerning the underpinnings of expertise.

We also offer one more specific recommendation for future research on expertise. The goal of Friedlander and Fine’s (2016) *grounded expertise components approach* (GECA) is to identify predictors of individual differences in expertise in a theoretically neutral manner to minimize bias in findings from research concerning the relative importance of one class of factor versus another (e.g., training vs. basic abilities). The approach begins with exploration: administering a survey to a large sample of performers in domains, with questions about their level of engagement in training activities, as well as education, interests, hobbies, careers, accomplishments, and other characteristics that may be informative about potential predictors of expertise (i.e., performance differences). The survey data are then analyzed to identify a candidate set of potential predictors of expertise. Finally, a study is conducted to estimate the relative contributions of the factors to the prediction of individual differences in expertise. Programmatic research can then proceed on this basis.

A major goal of the GECA is to identify activities that consistently correlate to a practically and statistically significant degree with measures of expertise. These activities may differ across domains. More specifically, for some domains, the activities may meet the ultimate criteria for deliberate practice, whereas for other domains, they may include unstructured activities that do not fit the definition of deliberate practice, purposeful practice, or even naïve practice. Furthermore, within a domain, there may be multiple “routes” to developing expertise. That is, one performer may achieve a given level of expertise by engaging in one set of activities, whereas another performer may achieve the same level of expertise by engaging in a different set of activities. As an illustration, Berliner (1994) found that jazz musicians emphasized the importance of intentionally unstructured “jam” sessions for developing improvisational skill, and noted that “[s]trongly motivated students commonly learned musical instruments without formal instruction by synthesizing bits of knowledge from commercial method books, other young performers, and their own experimentation” (Location 738).

In short, the search for activities using Friedlander and Fine’s (2016) GECA holds great potential to shed light on the role of different types of training in explaining expertise across a wide range of domains, including not only domains traditionally studied in expertise research (e.g., chess, sports, and classical music) but also those that have received relatively little attention in research.

## Conclusion

For decades, the field of expertise has focused on environmental factors as the major determinants of individual differences in expertise, whereas genetically influenced factors are assumed to play a relatively unimportant role, if any role at all (Ericsson et al., 1993; Ericsson, 2007a). Environmental factors certainly are important to consider in investigating the origins of individual differences in expertise, but a comprehensive scientific theory of expertise must take into account genetically influenced factors as well, including basic abilities and capacities (“talent”). At a more general level, we argue that it is time—*past* time—for the nature vs. nurture debate to be over in the field of expertise, as it has been in most areas of psychological research for decades (Turkheimer, 2000). Embracing the idea that expertise can be best understood as a product of gene-environment interplay (nature *and* nurture) will, as Plomin (2018) recently observed, move the field ahead and integrate it with the life sciences. At a practical level, findings from this research will provide a scientific foundation for principles and procedures designed and implemented to accelerate people’s acquisition of complex skills across a wide range of domains and elevate the performance of individuals, organizations, and societies.

## Reflection

As we were editing the page proofs for this article, we received the sad news of Anders Ericsson’s passing. It is difficult to imagine the field of expertise without Anders—he was a pioneer. But his ideas will live on and continue to inspire scholarship and debate that will lead to greater understanding of the subject about which he was so passionate. We hope that Anders found our work as stimulating as we found his. He forced us to think critically about our most basic assumptions concerning expertise, and to try to put our best case for our perspective forward. We are in his debt, and extend our sincere condolences to his family, friends, students, and colleagues.

## Author Contributions

DH was the primary author and conceived the article. BM and FO provided extensive comments and edits on multiple drafts. All authors contributed to the article and approved the submitted version.

## Conflict of Interest

The authors declare that the research was conducted in the absence of any commercial or financial relationships that could be construed as a potential conflict of interest.

## Figures and Tables

**FIGURE A1:**
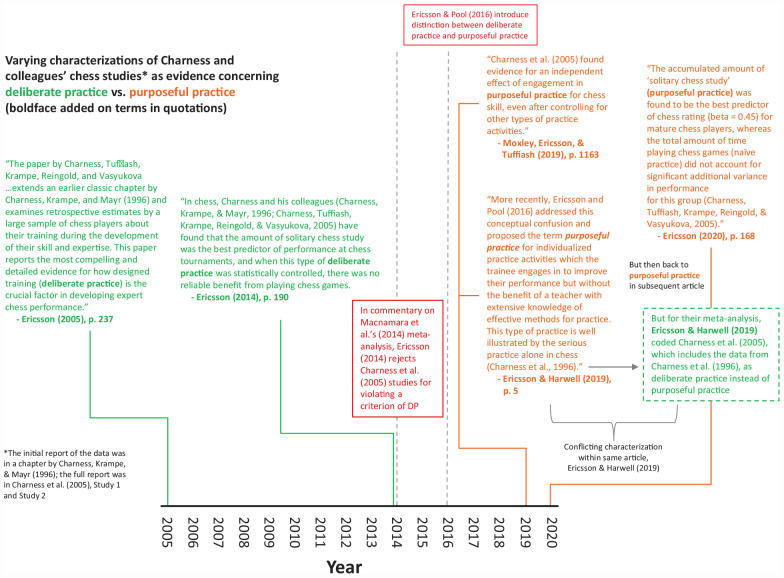
Varying characterizations of Charness et al. (2005) chess studies in Ericsson’s writings.

## Footnotes

1 http://www.world-memory-statistics.co.uk/disciplines.php

2 There were two studies in this project. Charness et al. (1996) is a chapter and provides an initial report of data from Study 1 (*N* = 136 of an eventual *N* = 239); Charness et al. (2005) is a journal article and reports the full results (Study 1 *N* = 239; Study 2 *N* = 169).

3 Ericsson and Harwell (2019) state they coded eight effect sizes as deliberate practice and six effect sizes as purposeful practice. They list the deliberate practice effect sizes being from: Baker et al. (2003), Charness et al. (2005), both effect sizes; Ericsson et al. (1993), both effect sizes; Schultetus and Charness (1997), Gobet and Campitelli (2007), and Ruthsatz et al. (2008). And they list the purposeful practice effect sizes being from: Duffy et al. (2004), Tuffiash et al. (2007), Harris (2008), Duckworth et al. (2011), and Maynard et al. (2014). We assume that De Bruin et al. (2007) was the sixth purposeful practice study and that its omission from the latter list is a clerical error by Ericsson and Harwell (2019).

4 Following Ericsson and Harwell’s (2019) terminology, when referring to all 14 effect sizes (six coded as purposeful practice and eight coded as deliberate practice) in the meta-analysis, we use the label “purposeful/deliberate practice.”
